# Exploring shared risks through public-private partnerships in public health programs: a mixed method

**DOI:** 10.1186/s12889-017-4489-z

**Published:** 2017-06-12

**Authors:** Wadi B. Alonazi

**Affiliations:** Health Administration Department, College of Business Administration, King Saud University, PO Box 71115, Riyadh, 11587 Saudi Arabia

**Keywords:** Public-private partnerships, Vision 2030, Risk shared, Saudi national transformation program, Public health programs

## Abstract

**Background:**

The natural assimilation of the process through which health partners sustain long-term relationships is a key issue in maintaining social well-being, reducing health risk factors, and sustaining public health programs. One global initiative in building effective healthcare systems is public-private partnerships (PPPs). This study elucidates the proposed key performance indicators initiated by the Ministry of Health of Saudi Arabia based on the projections of the government, known as Vision 2030, from the perspective of health risk factors.

**Methods:**

Through an inductive content analysis, this study assessed primary and secondary data in relation to the Saudi National Transformation Program (NTP). To identify the institutions that played a role in formulating the new Saudi Healthcare System, health policies, regulations, and reports published between 1996 and 2016 were categorized. After ranking the risk factors, the investigator selected 13 healthcare professionals in four focus group interviews to insightfully explore the challenges that the NTP faces from a health risk perspective. Thus, the study employed qualitative data gathered through focus group interviews with key figures as well as data extracted from written sources to identify distinct but interrelated partnerships practiced within risk management.

**Results:**

A methodological overview of NTP priority and implementation offered practical guidance in the healthcare context. The five critical factors in maintaining successful and sustainable PPPs were (1) trustworthiness, (2) technological capability, (3) patient-centeredness, (4) competence, and (5) flexibility. Concession on primary and secondary healthcare services might be a good option based on the literature review and considering its popularity in other countries. A high outcome-based risk of PPPs was found as the most commonly shared perspective in risk management.

**Conclusions:**

Although the impact of the NTP rise has yet to be explored, its potential for challenging health consequences requires consideration and substantial regulatory action. This study contributes to the emerging critical analysis on local health initiatives by highlighting how integration may only be possible with a more radical conceptualization of national health governance.

## Background

For decades, the Ministry of Health (MoH) of Saudi Arabia (SA) has substantially financed and delivered 60% of the Saudi Healthcare System (SHCS), although other semi-governmental agencies and private sector actors have had their equal share of influence. Except for three landmark events—launch of the Council of Cooperative Health Insurance (CCHI) in 1999, Saudi Central Board for Health Accreditation Institutions (CBAHI) in 2005, and Saudi Health Council (SHC) in 2009—the SHCS has not changed significantly in terms of major financing and regulation [1–3]. The Saudi constitution emphasizes effective and accountable healthcare provisions for the entire population through universal access to basic healthcare services. Although the government subsidizes the public healthcare system, the system’s performance level has not yet shown promise, especially competent medical specialization [4]. Various forces have challenged the government to improve the quality of care, but there is a reluctance to dramatically intervene in SHCS components. Apparently, Saudi healthcare leaders have already decided to improve the system, bearing some risks [5].

The Saudi Arabian government proposed a National Transformation Program (NTP) in 2016 due to leadership succession, a drop in oil prices, and rapid medical development and practice. The NTP requires that each public institution improve its performance by gradual cooperation between public and private partnerships, known as public-private partnerships (PPPs) [6]. Thus, the MoH initiated 15 key performance indicators (KPIs) to meet this objective. While the MoH liaises with some private sector entities to treat individual cases locally, the private sector may not offer comprehensive treatment without financial support from a third party or self-payment. Such cases indicate inequality in the delivery of treatments, such as financial concession and disease management, indicating that the poor cannot access necessary treatments due to lack of financing. Under the NTP, primary and secondary healthcare organizations, whether public or private, must cooperate to provide basic healthcare services within a PPP framework. Unfortunately, much of the inequality in the SHCS may persist in non-hospital provisions, especially social services [7]. Therefore, PPPs have received significant attention in the healthcare system, and organizing and funding the system are only a small part of the research agenda that still need to be determined [8, 9]. Simply, the policy towards PPPs should be effective, comprehensive, and implemented over the course of time with deadlines [10]. This study, through a holistic approach, sheds light on the proposed KPIs initiated by the MoH, in which health risks are minimized and the system performs efficiently based on the NTP through engaging PPPs.

### Public- private partnership

According to the World Bank, PPP is ‘a long-term contract between a private party and a government entity, for providing a public asset or service, in which the private party bears significant risk and management responsibility, and remuneration is linked to performance’ [11]. This form of partnership involves a wide range of activities that vary based on the extent of involvement and the unexplored risks taken by both public and private parties [12]. The SHCS has been practicing individual referrals to many national and international health institutions through its ‘direct-payment’ system, which exceed $16 billion for approximately 2000 overseas cases and 60,000 local cases annually; however, the NTP may exceed 20 million cases [13].

To maintain its economic growth, the government has set aside roughly $10 billion to invest in PPPs, primarily by encouraging private sector investment and participation at all levels of healthcare provision. To optimize the efficiency of the private sector and effectiveness of the public sector, the NTP proposed that the MoH transform the healthcare system gradually and in stages while employing PPPs. These stages are based on a stewardship model in which the private sector takes more of a partnership role in certain aspects of the process to accomplish the mission of a project, whereas the public sector assesses the process based on certain KPIs. Consequently, there is high risk to both parties in terms of finance and operations. Using a partnership taxonomy based on the appropriate structure, process, and outcomes model, the healthcare system may inclusively identify three types of PPPs, as illustrated in Table 1 [14].

**Table 1:** Common risk ranking within the PPP framework

Risk Ranking	Structure	Process	Outcomes
Low	DBFo	bOo/OM	boT
Intermediate	BLT/L	BTO	bOoT/bLOT/bLTM
High	LDO	LORT/JV	Concession

Note: *D* Design, *B* Build, *F* Finance, *o* Own, *b* Buy, *O* Operate, *M* Maintenance, *T* Transfer, *L* Lease, *JV* Joint venture, and *R* Rehabilitate

In this study, shared risk is defined as the structured cooperation between public and private sectors to share clinical and non-clinical consequences for individuals [15]. Normally, a risk is shared on three levels: public, private, and patients [16]. This taxonomy focuses on facilitating the levels of risks within common types of PPPs, characterized by a close working partnership between public and private institutions. Risk ranking management at the national level requires ranking risks according to defined standards. Such standards usually take the form of healthcare system performance, especially performance in eliminating disease burdens. The standards also contribute to specifying the level of performance from each party, although they also consider risk occurrence, severity, and error detection [17].

Table 1 allows the reader to rank the evidence on the structure, process, and outcome levels in a hierarchy by identifying ‘input’ as a legal structured risk, ‘process’ as an intermediate process risk, and ‘concession’ as a high outcomes risk. This taxonomy modifies both parties by means of varying channels. First, ineffective communication and absence of leadership in complex organizations may reduce their overall performance and keep the organizations from accomplishing their goals; therefore, to be effective, organizations may collaborate in the form of partnerships [18]. Due to increased complexity, the need to collaborate is especially relevant to organizations that work with emerging technologies. These technologies are at the forefront of knowledge, and various sectors need to share knowledge to develop technologically [19]. Another business-point reason why organizations form PPPs is that a group of organizations is better poised to overcome market deficiencies than a single organization, especially in medical technology [19–21].

Potentially, shared risk can fit within public and private institutions [22]. If the notion of a private provision, is to run ‘low risk’ services and that of a public provision is to run services with ‘substantial risk’, how can the share of risk be equal? Although this approach has been implemented widely, governments have relied heavily on private industry to provide supplies and services for many healthcare activities, from infrastructure to hospital performance measurement. The private sector is underutilized in providing healthcare services; however, the risk may be shared in SHCS through major healthcare indicators shown in Table 2 [13, 23].

**Table 2:** Major health demographic indicators in 2016

Indicator	Data
Total population (million)	31.00
Citizens (million)	20.00
Mortality rate under 5 (per 1000)	15.20
Population growth (annual %)	2.11
Birth rate, crude (per 1000 people)	22.00
Death rate, crude (per 1000 people)	4.00
Life expectancy at birth, total (years)	74.00
Total fertility rate (births per woman)	3.00
Fertility rate (births per 1000 women aged 15–19 years)	18.00
Urban population (% of total)	84.00

### Healthcare economics

Based on the population’s socioeconomic indicators, health economics is an essential principle governing the performance of health organizations [24]. The traditional role of public institutions is developing public health and social welfare through not-for-profit health organizations, while private institutions aim to increase profit but through less risky performance [25]. Saudi Arabia’s economy is heavily based on its natural oil reserves; therefore, the budget is normally associated with increases or decreases in oil prices in the fossil fuel market. From the end of 2015 and all through 2016, there has been a substantial decrease in crude oil revenue worldwide. Consequently, this has led to a significant decrease in government funds allocated to the MoH budget in 2016, as compared with 2015 (−34.5%).

As the MoH intends to propose serious steps to reengineer the SHCS through the NTP, the proposed initiative aims to reduce costs by, on one hand, sharing risks between public and private institutions and, on the other hand, engaging community-driven approaches within the healthcare system [26, 27]. The current Saudi system is under significant pressure, primarily because of the inappropriate utilization of the rising proportion of provincial budgets and, secondarily, because of the below-intermediate quality of care provided to the population versus high government expenditure [28, 29]. Due to high costs, emerging advanced technologies are prevalent indicators of public economic growth; however, in Saudi Arabia, such technologies are rarely available within the private sector [30].

### Regulating the healthcare system

One central component of any effective healthcare system is its policies. Administrative efforts underpin the movement of health institutions and gradually govern patient flow. Therefore, any health inequality is attributable, at least in part, to administrative deficiencies [31]. Surveying a given situation requires epidemiological analysis to identify risk factors for particular diseases, such as infectious diseases, and targets for health regulations. Measurability has become a key determinant of health outcomes, and such KPIs are leading factors in either weakening or strengthening the SHCS. Political and organizational approaches to policy analysis estimate the policy itself as a decision-making process rather than focusing on health output [32]. Measuring patient behaviours and endorsing ineffective health awareness and misled ideologies in medicine were two common themes within a primarily publicly funded system [33–35]. Even so, effective regulation and government support were the key elements of national health progress [36]. This study reports on details of how the surrounding risks might affect PPPs.

## Methods

This study uses a mixed-method approach utilizing both qualitative and quantitative domains to explore the NTP, provide details of PPPs, and focus on more features and models to explain effective PPPs, respectively. To share risks, this study attempts to clarify the PPP process and explain its integration within a universal healthcare system.

### Procedure

Initially, this study organized common health policies issued by the relevant agencies between 1996 and 2016 based on risk factors associated with the role of each institution, that is, on the primary and secondary levels. For the qualitative approach, this study analysed semi-structured interviews while employing focus group.

First, the author reviewed major health policies and reports issued in the decades after 1996. This included articles issued by the CCHI, CBAHI, and the SHC. Moreover, the study analysed the initiative proposed by the MoH to meet Vision 2030 through the NTP. This stage involved identifying the role and implication of each public and private institution. Then, the risk was ranked subjectively based on the risk level faced by the components of the healthcare system. The study describes the major domains based on expert reviews that may streamline the transformation within the SHCS. The interview went through two stages. Initially, all participants were asked about major domains that the SHCS may need to meet future challenges. The four focus group interviews enabled the development of relationships among the sample. Finally, each group rationally ranked these domains, highlighting their implications and impact on the SHCS. Thus, triangulation assures that this study captures the different levels and dimensions of the PPPs as the relationship among key constructs can be identified and empirically documented.

By discussing the role of public and private institutions, the aim of the review was to look for patterns rather than test or confirm a hypothesis. The focus was on gaining insight and familiarity regarding the PPP areas for a more rigorous investigation while utilizing a mixed-research methodology. After obtaining ethical approval, the researcher approached a workshop supported by the MoH to prepare for the inauguration of Vision 2030. The participants were interviewed collectively to determine several domains that may govern the SHCS. Then, individually, each member was assigned a rank for each domain, indicating its priority. To ensure validity, the potential homogenous sample indicated that the collection of new data did not shed any further light on the subject under investigation.

### Sample

Known of their conceptual and practical knowledge of the PPPs, the purposive sample consists of 13 healthcare professionals belonging mainly to the most representative segments of the SHCS workforce and those who have effectively participated in continuous workshops held by the MoH. The researcher selected this sample mainly due to the participants’ respective leadership roles in developing MoH initiatives. These initiatives were planned by the MoH and a panel of 30 experts proposed different methods in addressing Vision 2030.

### Data analysis

Data relevant to the research questions were obtained mainly through major resources, such as the rules and regulations issued by the MoH, Council of Ministers memos, and guidelines enforced by public and private organizations. The process of identifying statements that support and describe the function of the institutions was developed based on the roles and implications involved. Furthermore, the functions were based on the regular impact on providers and categorized into different levels. For the quantitative approach, major domains were formulated according to four experts’ responses.

Credibility and transferability were used to infer trustworthiness and to reduce the effect of investigator bias. One way to achieve this was to seek domain agreement in the first stage with the second stage among experts. Again, in the second stage, findings had been transferred to the focus group. This may increase the trustworthiness of the most probable interpretations.

## Results

For the qualitative approach, the fundamental roles of public and private institutions that are significantly attributed to maintaining the relationships among themselves were identified, as shown in Table 3.

**Table 3:** Roles and functions of major institutions attributed to the success of the NTP

Institution	Mission	Implication	Institution Type	
			Public	Private
CBAHI	Enforcing quality	High	Restricted	Unrestricted
CCHI	Financing roles	High	Unrestricted	Restricted
SHC	Regulating/coordinating	Law	Restricted	Unrestricted

Note: Public institutions aim to reduce risk by improving the quality of care, incurring financial support from the government, and coordinating with the SHC to refer patients.

Table 3 summarizes the roles and functions of major institutions associated with the success of the NTP. Previously, the government financed and regulated the healthcare system; however, the private sector may now interfere and regulate the healthcare system.

An aspect of PPP is to identify public and private sectors that can collaborate to improve the SHCS in the future. Table 4 presents a cohort analysis of the major indicators of both public and private sectors (2011–2016).

**Table 4:** Health indicators based on PPP provisions

Provisions	2011	2012	2013	2014	2015	2016
Public hospitals	246	251	259	268	270	275
Private hospitals	130	137	136	141	145	147
Public PHC	2109	2259	2259	2281	2282	2300
Private PHC	1987	2168	2249	2408	2670	2710

**Fig. 1:**
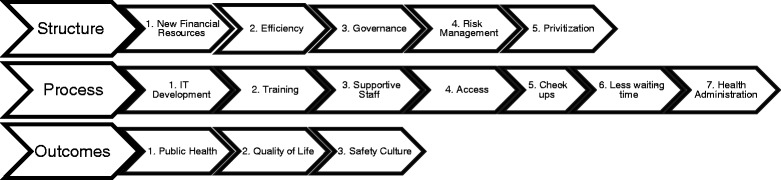
Summarizes the 15 initiatives proposed by MoH along with the structure, process, and outcomes model

The MoH has recently proposed rigid KPIs to instil PPPs gradually by the end of 2030, as shown in Fig. 1. The proposal promotes a strong health economy at both preventive and curative levels, and the spread of KPIs will massively impact the health labour market and skills needed to develop social welfare. Although the full potential of improving health through such KPIs is yet to be discovered, the proposed model is developing policy and supporting research to make people healthy for life and work in a rapidly technologically developing society by 2030.

Among the 15 MoH initiatives, almost half (47%) are process-based measures, while the rest are either structure- or outcomes-based measures (33% and 20%, respectively).

Based on the analysis, there is a new trend of healthcare prioritizing and implementing, which has yielded in unclear results for addressing the challenges faced by PPPs. Table 5 summarises the priorities and challenges in healthcare based on the answers of experts involved directly in implementing the health transformation. The aim of this group was to provide reliable, comparable qualitative data about the future of PPPs, based on thematic analysis.

**Table 5:** Priorities and challenges for the SHCS

Domain	Rank	Physicians (*n* = 2)	Nurses (*n* = 5)	Administrative Staff (*n* = 4)	Technicians (*n* = 2)	%
Trustworthiness	1	2	5	4	2	100
Technology	2	0	5	4	2	84
Patient-centeredness	3	1	3	4	2	76
Competence	4	1	3	3	1	61
Flexibility	5	0	2	2	1	38

The 13 participants believed that the SHCS exhibits a need for more trustworthiness between the public and private sectors to achieve success. Second, they presumed that the technology and patient-centeredness domains are key issues in successful partnerships. Having less importance than previous factors, healthcare professionals’ competence and flexibility within the healthcare system were identified as obstacles that PPPs may face.

## Discussion

The purpose of a health model is to decrease potential personal and organizational risk factors associated with implementing the NTP. The integration of health reports and indicators may lead to a better understanding of healthcare priorities in coming decades [37].

Organizationally, there are some concerns regarding the progress of quality culture within the majority of the primary and minority of the secondary healthcare services, the proposed model may yield to both regulatory and economic obstacles unless the implementation phases guarantee an injection of capital [1–3, 36]. Risk is normally associated with both regulation and financing of the SHCS. Based on the results, in order to gradually introduce the role of the private sector and retain the finances of the public sector, it is imperative to consider ‘concession’ as one of the most suitable risk-shared indicators while delivering the SHCS through PPPs, especially in the case of primary and secondary healthcare services [38]. This is a widely practiced process, especially in the United Kingdom and Australia. Thus, the CCHI, CBAHI, and the SHC should no longer play small roles in the healthcare system but instead employ insightful curative health services while building disease-management and drug-regulation mechanisms [39]. Indeed, the MoH may also still operate in a regulative position, but only with limited influence in operation and maintenance. Thus, the 15 MoH initiatives will measure the outcomes of each provision, and reimbursements could be based on performance. To form a mutual strong attribution of PPPs, public and private sectors should set preliminary trust of relationships, especially in reimbursement.

A trust-based model can dynamically facilitate the demands of patients and the activities provided depending on the proposed reimbursement system [40]. Under the NTP, the PPP program clearly shapes the values towards health services and their financing arrangements. For public primary healthcare services, the development of medical technology is slow, unlike for private primary healthcare services. Contrarily, secondary private healthcare services lack advanced technology. Nowadays, providing effective public services and addressing the risk balance are basic requirements measured by third parties and regulatory institutions [41]. There are also intangible rewards at play, such as the healthcare reputation of the institution or physician/specialist at hand. As patient-centeredness is one of the six domains for any effective healthcare system, the SHCS should incorporate the voice of the patient in its overriding goals [17, 42]. In this study, the domain that had the most difficulty in incorporating PPPs was flexibility. Organizational flexibility refers to institutions enabling patients to process their treatments without complications, as a potential management strategy [43]. Thus, the goals must include ensuring adequate and continuous usability of the facility over time, providing amenities, and supporting/creating high-quality services.

As public investment in infrastructure continues to decrease, private participation is required to address the existing and expanding deficit in medical services, especially for chronic cases, patient behaviour, and treatment mechanisms [35]. Despite great progress in PHC services, PPPs may be more successful in secondary healthcare services due to high demand from the population.

This study has some limitations including understating the exact roles of both public and private sectors. This study has not tackled the nature of information shared between the PPPs and NTP. Thus, additional means of improving healthcare, especially when introducing private roles in healthcare services, may yield inequalities in cases with high costs [27]. There have been some concerns in building effective PPPs attributable to the period of transformation during which the study occurred. Additionally, the current NTP has not yet addressed the reimbursement system. For future research studies, concentrating on the possible impact of each initiative may be helpful in understanding and measuring the effective achievement of each domain of the program.

## Conclusions

This paper explores shared risks through PPPs in public health programs, and suggests models for public health policy makers when implementing the NTP. The most appropriate model to gradually implement the NTP is through concession based on primary and secondary treatment. Additionally, CCHI, CBAHI, and SHC should take more active roles in regulating the SHCS. Finally, to ensure effective healthcare system, relationships between public and privet sectors should be tightened based on utilizing high technology including the voice of the patient, increasing competency, and ensuring flexibility.

## Abbreviations

CCHI: Council of Cooperative Health Insurance
KPIs: Key Performance Indicators
MoH: Ministry of Health
PHC: Primary Healthcare Center
PPPs: Public-Private Partnerships
SA: Saudi Arabia
CBAHI: Saudi Central for Health Accreditation Institutions
SHC: Saudi Health Council
SHCS: Saudi Healthcare System
NTP: Saudi National Transformation Program
